# Rationalisation of the Differences between APOBEC3G Structures from Crystallography and NMR Studies by Molecular Dynamics Simulations

**DOI:** 10.1371/journal.pone.0011515

**Published:** 2010-07-12

**Authors:** Flavia Autore, Julien R. C. Bergeron, Michael H. Malim, Franca Fraternali, Hendrik Huthoff

**Affiliations:** 1 Randall Division of Cell and Molecular Biophysics, King's College London, London, United Kingdom; 2 Department of Infectious Diseases, King's College London, London, United Kingdom; Institut Pasteur Korea, Republic of Korea

## Abstract

The human APOBEC3G (A3G) protein is a cellular polynucleotide cytidine deaminase that acts as a host restriction factor of retroviruses, including HIV-1 and various transposable elements. Recently, three NMR and two crystal structures of the catalytic deaminase domain of A3G have been reported, but these are in disagreement over the conformation of a terminal β-strand, β2, as well as the identification of a putative DNA binding site. We here report molecular dynamics simulations with all of the solved A3G catalytic domain structures, taking into account solubility enhancing mutations that were introduced during derivation of three out of the five structures. In the course of these simulations, we observed a general trend towards increased definition of the β2 strand for those structures that have a distorted starting conformation of β2. Solvent density maps around the protein as calculated from MD simulations indicated that this distortion is dependent on preferential hydration of residues within the β2 strand. We also demonstrate that the identification of a pre-defined DNA binding site is prevented by the inherent flexibility of loops that determine access to the deaminase catalytic core. We discuss the implications of our analyses for the as yet unresolved structure of the full-length A3G protein and its biological functions with regard to hypermutation of DNA.

## Introduction

The human APOBEC3G protein (A3G) is a cellular polynucleotide cytidine deaminase that can restrict the spread of HIV-1 in the absence of the viral Vif protein [1]. HIV-1 Vif specifically interacts with A3G and targets it for proteasomal degradation, thereby lifting the barrier that A3G poses to virus replication [2], [3]. In the absence of Vif-mediated down-regulation, A3G is packaged into assembling virions where it interferes with the elongation of reverse transcription and catalyses deamination of cytidine to uridine in nascent reverse transcripts [4]–[9]. This causes the progeny viruses that have been exposed to A3G to lose infectivity by virtue of halted reverse transcription and the loss of genomic integrity. In addition to its activity as an HIV-1 restriction factor, A3G as well as other APOBEC proteins can inhibit the spread of several other viruses and transposable elements [10].

The APOBEC3G protein contains two cytidine deaminase (CDA) domains, termed the N-terminal and C-terminal CDAs (N-CDA and C-CDA, respectively). The CDA domains of all APOBEC3 (A3) proteins are predicted to fold into a mixed five-stranded β-sheet that exposes on one face two α-helices that contain the H/C-X-E-X_23-28_-P-C-X_2_-C zinc coordination motif [11]. This zinc-coordination motif is essential for catalysis of the deamination reaction, yet not all the zinc-coordination motifs encountered in A3 proteins constitute an active deaminase catalytic core. In particular, the N-CDA of A3G is catalytically inactive but is required for virus inhibition by mediating RNA-dependent oligomerisation and packaging into virions [12]–[17]. Conversely, cytidine deamination is strictly mediated by the C-CDA of A3G [10], [18]–[20].

The deamination activity of A3 proteins is also commonly referred to as DNA editing, in reference to the founding member of the APOBEC protein family, APOBEC1 (A1), which is an RNA-editing enzyme [21], [22]. Unlike A1, A3G is unable to edit RNA and its editing activity is tightly restricted to single stranded DNA substrates. A3G preferentially edits the third cytidine in a 5′-CCC-3′ trinucleotide sequence context on single stranded DNA, whereas it is unable to edit double stranded DNA or DNA/RNA hybrids [6], [23], [24]. The selective editing of single stranded DNA is a shared property of A3 proteins, although differences in target site preferences do exist. For example, APOBEC3F (A3F), another member of the A3 family that restricts HIV-1 in the absence of Vif, preferentially edits in a 5′-TTC-3′ sequence context [25]–[27].

Although structural data on the full length A3G protein are currently lacking, several recent studies have reported high-resolution structures of truncated A3G constructs containing the catalytic C-CDA moiety of A3G (table 1). First, Chen et al. reported the solution structure of a 187 amino acid fragment by NMR, encompassing amino acids 198 up to 384 of A3G, which contained 5 mutations that were introduced to enhance the solubility of the protein (PDB code 2JYW) [28]. Second, Holden et al. obtained crystals that diffracted to a resolution of 2.3 Å with a 184 amino acid fragment, encompassing amino acids 197 up to 380 of A3G with the wild type sequence (PDB codes 3E1U and 3IQS) [29]. Third, Furakawa et al. reported the NMR structure of a 192 amino acid fragment, encompassing amino acids 193 up to 384 of A3G, also with the wild type sequence (PDB code 2KBO) [30]. Fourth, Harjes et al. reported an NMR structure of a 194 amino acid fragment encompassing residues 191 up to 384, which also contained the five solubility enhancing point mutations (PDB code 2KEM) [31]. Finally, a crystal structure of the 191–384 fragment containing the solubility enhancing mutations at a resolution of 2.25 Å has been reported most recently (PDB code 3IR2) [32]. For clarity, the original references and accession codes are shown in table 1, along with the nomenclature we will adopt from this point on.

**Table 1:** Nomenclature of APOBEC structures used in this study with references.

Nomenclature	PDB code	Reference
NMR1-2K3A	2JYW	Chen K.M., *et al.* Nature (2008) 452:116–9
NMR2	2KBO	Furukawa A., *et al.* EMBO J. (2009) 28:440–51
NMR3-2K3A	2KEM	Harjes E., *et al.* J Mol Biol (2009) 389:819–32
XRAY1	3E1U*/3IQS	Holden L.G., *et al.* Nature (2008) 456:121–4
XRAY2-2K3A	3IR2	Shandilya S.M.D. *et al.* Structure (2010) 18:28–38
A2	2NYT	Prochnow C., et al Nature (2007) 445: 447–451

* The crystal structure originally deposited as 3E1U was found to contain an error with regard to fitting of residue W269 to the electron density. This was corrected in the structure deposited as 3IQS. The positioning of W269 in 3E1U and 3IQS does not affect the conformation of β2 or the proposed DNA binding pocket. MD simulations described here were performed with 3E1U.

**Figure 1:**
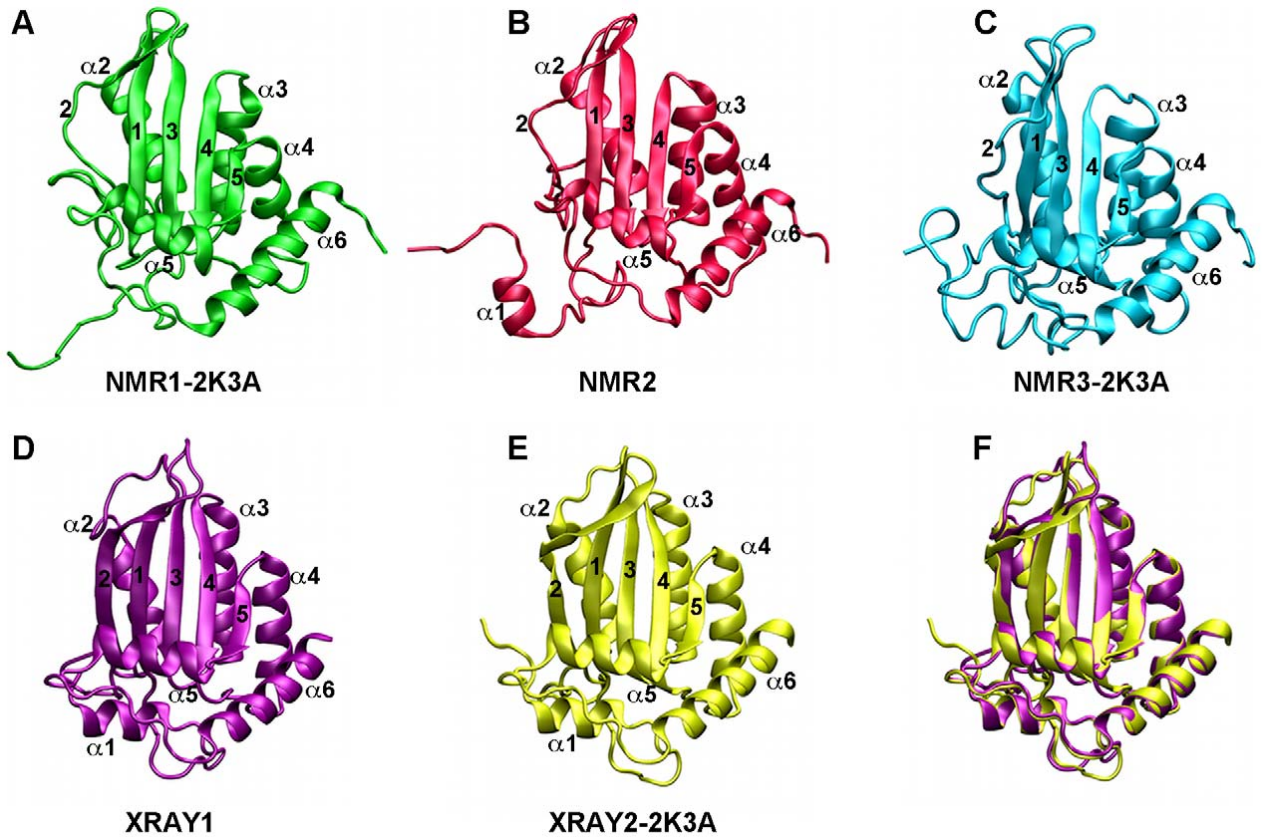
Ribbon representation of high-resolution structures of the A3G C-CDA. (A) Structure of NMR1-2K3A shown in green. (B) Structure of NMR2 shown in red. (C) Structure NMR3-2K3A shown in blue. (D) Structure of XRAY1 shown in purple. (E) Structure of XRAY2-2K3A shown in yellow. (F) Superimposition of XRAY1 and XRAY2-2K3A. The colour coding is maintained throughout. Details of these structures are described in the text and the original references as well as the nomenclature adopted here are given in table 1. β-strands are numbered 1 through 5 and α-helices are numbered α1 through α6.

Throughout these studies most of the core secondary structure elements that characterise APOBEC proteins, as first reported in the crystal structure of human APOBEC2 (A2)[33], were preserved. However, a number of differences between these structures are also apparent. Most strikingly, a β-strand at the edge of the mixed sheet, termed β2, is predominantly disordered and discontinuous in all NMR structures, regardless of the presence or absence of the solubility enhancing mutations (Figure 1). The β2-strand is much more defined through interactions with the β1 strand in the crystal structure with the wild type sequence, XRAY1, although even in this structure a small distortion from regular β-sheet geometry is apparent due to the bulging out of a single amino acid, Q237. The β2-strand from the crystal structure with mutations, XRAY2-2K3A, is also considerably more defined than in the NMR structures, but in this case the bulge contains amino acids N236, Q237, R238 and R239 (Figure 1).

The conformation of the β2 strand has important implications for the folding of the full length A3G protein [12], [34]. The full-length A3G polypeptide consists of two CDA domains, but it remains unknown how these domains are positioned relative to each other. We and others have previously proposed homology models of A3G in which the N- and C-terminal CDA domains are aligned via interactions involving β2 to form a continuous β-sheet as observed in the crystal structure of the closely related A2 protein [12], [16], [35]. This continuous sheet would be consistent with the conformation of β2 as observed in the first crystal structure of the A3G C-CDA, but the distorted β2 observed in the NMR structures and the second crystal structure may interfere with this arrangement (Figure S1).

There is also disagreement with regard to the assignment of residues within the A3G C-CDA that participate in interactions with the substrate DNA. To date, no structure of the A3G protein bound to DNA has been obtained and the identification of residues that interact with the substrate has therefore relied on indirect methods. Importantly, DNA-binding grooves proposed on the basis of the wild-type crystal structure [29] and the first reported NMR structure with mutations [28] have entirely different orientations [34]. Furthermore, empirical identification of amino acids that interact with the DNA by chemical shift perturbations in response to DNA titrations have yielded significantly different sets of candidate residues in two NMR studies [28], [30]. Positioning of loops in the vicinity of the catalytic core also differs significantly in all of these structures (Figure 1), with the two crystal structures sharing the highest overall similarity (table 2 and Figure 1F).

**Table 2:** RMSD of Cα atoms for the reported high-resolution structures of the A3G C-CDA.

	NMR1-2K3A	NMR2	NMR3-2K3A	XRAY1	XRAY2-2K3A
NMR1-2K3A	0				
NMR2	2.985	0			
NMR3-2K3A	4.689	4.220	0		
XRAY1	4.621	4.348	3.017	0	
XRAY2-2K3A	4.726	4.228	3.004	1.814	0

We note that the differences between the two crystal structures appear to be mostly due to different crystal packing interactions. In the XRAY1 crystal, the C-CDA is clearly observed as monomeric within the unit cell [29], whereas the unit cells of the XRAY2-2K3A crystal contain dimers of the C-CDA [32]. Most of the solubility enhancing mutations present in XRAY2-2K3A are in close proximity to the multimer interfaces observed in that crystal (not shown), suggesting that these mutations may have affected the crystal packing. Although it has been suggested that the A3G C-CDA has the capacity to oligomerise [36], none of the interfaces observed in the XRAY2-2K3A crystal have been demonstrated to be of significance for biological activities [32]. The monomeric state of the A3G C-CDA as observed in the XRAY1 structure is furthermore supported by ultracentrifugation analyses that were performed on the NMR1-2K3A preparation [28].

Molecular dynamics (MD) simulations are a computational means to probe molecular motions at an atomic scale and provide the opportunity to monitor dynamical features of protein structures that are not readily obtainable from crystallography and NMR data. Here, we report MD simulations of each of the available high-resolution structures of the A3G C-CDA domain. The data sets from the MD simulations were specifically analysed to shed light on the uncertainties regarding the possible conformations of β2, as well as assignment of the DNA binding site.

## Results

### Antiviral and editing properties of A3G with solubility-enhancing mutations

**Figure 2:**
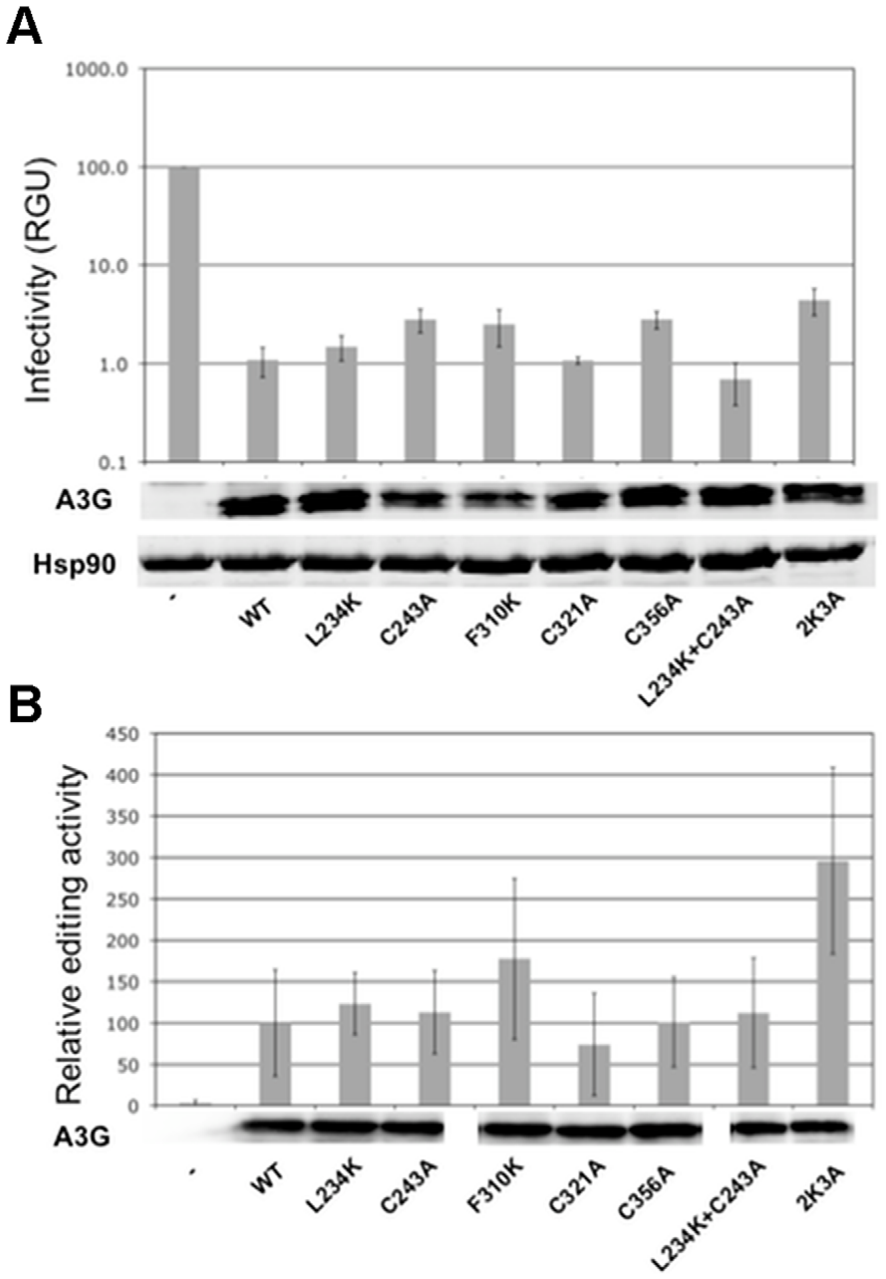
Virus inhibition and DNA editing by A3G with and without solubility-enhancing mutations. (A) Single-cycle infectivity of HIV-1/Δ*vif* viruses produced in the presence of wild type or mutant A3G measured in β-Galactosidase units (RGU) and presented as percent infectivity relative to the sample without A3G. Expression of wild type and mutant A3G proteins in 293T producer cells as determined by immunoblotting is shown beneath the graph. (B) Relative editing activity of wild type and mutant A3G proteins from 12 independent experiments. Immunoblots beneath the graph show the expression of A3G in equal volumes of bacterial cultures.

Because the structures reported by Chen, Harjes and Shandila all contain five mutations to enhance solubility of the protein [28], [31], [32], we first sought to determine whether these mutations alter the biological properties of A3G. Specifically, the truncated A3G constructs used in those NMR and crystallography studies contained the following mutations, which are collectively known as 2K3A: L234K, C243A, F310K, C321A and C356A (Figure S2). We assessed the effect of these mutations on antiviral activity and DNA editing, both individually and in combination. Because the L234K and C243A mutations are both located within β2 that appears distorted in these structures, we also constructed a mutant A3G containing both these mutations. We note that Harjes et al. already reported normal antiviral activity of full length A3G containing all five mutations (A3G-2K3A) with a C-terminally GFP-tagged A3G. Because we have previously observed that a GFP-tag on A3G can reverse the effect of mutations that cause reduced protein expression (Huthoff, unpublished results), we performed our mutational analysis with untagged full-length A3G. Single cycle infectivity experiments confirmed that inhibition of Vif-deficient HIV-1 by A3G was maintained in the presence of these mutations and that all proteins were expressed in similar amounts as determined by immunoblotting (Figure 2A).

We next assessed the DNA editing properties of the wild type and mutant A3G using a bacterial mutator assay [37]. Again, DNA editing by these mutant A3G proteins has previously been reported in the context of 191–384 and 198–384 truncated constructs [28], [31], but not in the context of the full-length protein. We observed wild-type levels of DNA editing with all constructs except the 2K3A-A3G, which displayed DNA editing activity that was approximately 2-fold higher than the wild type A3G (Figure 2B). These observations are entirely consistent with the previously reported studies with tagged or truncated A3G [28], [31], and indicate that A3G containing the solubility enhancing mutations maintained virus inhibition and DNA editing activities in the context of the full-length protein.

### The β2 region of the A3G C-CDA contains an insert of two amino acids

**Figure 3:**
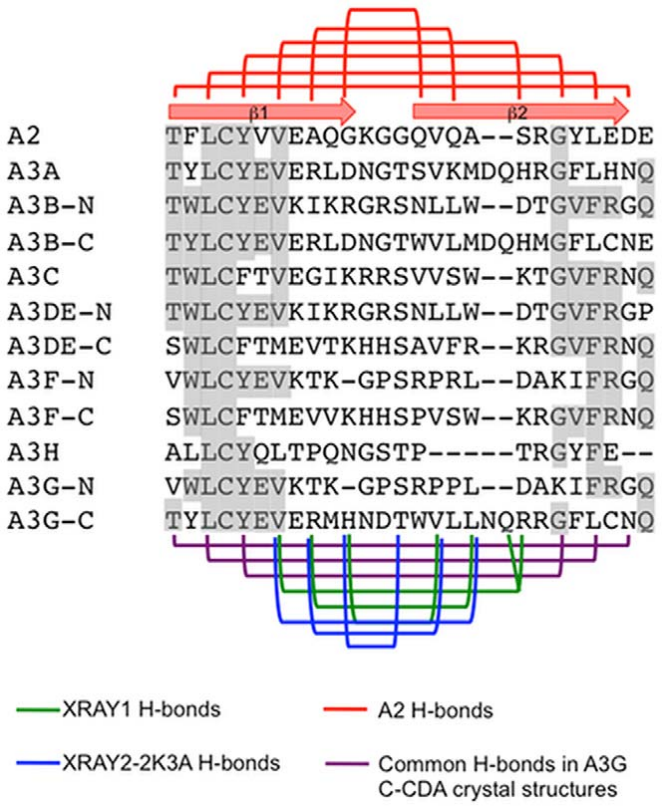
Alignment of the β1-β2 region from A2 and A3 proteins. Amino acid sequence alignment of the β1-β2 region from the human A2 and A3 proteins as generated with T-coffee, with the most conserved residues indicated by grey shading. The positions of β1 and β2 as present in the A2 crystal structure are indicated by arrows above the alignment. H-bonds between back-bone atoms in the β1-β2 sheet of A2 are indicated with red lines. Purple lines indicate H-bonds shared by the two crystal structures of the A3G C-CDA domain. The following H-bonds indicated by purple lines are also present in the NMR structures: L220-L242 in NMR1-2K3A and NMR3-2K3A; Y222-G240 in all NMR structures. Green lines indicate H-bonds unique to XRAY1 and blue lines indicate H-bonds observed in XRAY2-2K3A. Except for the absence of H-bonds between Y225-L235 in NMR2 and H228-T231 in NMR3-2K3A, H-bonds indicated by blue lines are also present in the NMR structures.

The distorted conformation of the β2 that is observed in the majority of A3G C-CDA structures represents a unique feature among CDA enzymes, as all other structures of these proteins show a defined and continuous β2 strand [34]. Indeed, the structure of the closely related A2 protein also shows an intact β2 region that is continuous with β1 and furthermore supports oligomerisation via β2-β2 interactions [33]. To determine whether this difference may be due to divergent primary sequences, we generated an alignment of the β1-β2 region from A2 and the human A3 proteins, indicating the β1-β2 interactions observed in the crystal structures of A2 and the various structures of the A3G C-CDA (Figure 3). From the alignment, it is apparent that the sequence of the β1 region contains a strongly conserved L-C-F/Y motif, which in the A2 structure interacts with a G-Y-L motif in the β2 region of A2. The latter motif is partly conserved in the human A3 proteins, and in A3G corresponds to G-F-L at positions 240 to 242. Importantly, the interaction between these motifs is evidenced by both crystal structures and is to variable extents also evident from the NMR data (Figure 3).

Throughout the remainder of the β1-β2 region, significant differences in the sequence between A2 and the A3 proteins arise, which in the case of A3A, A3B C-CDA and the A3G C-CDA includes a two-amino acid insert. It is in the direct vicinity of this insert, which in A3G corresponds to N236 and Q237, that differences between the H-bonding in the β1-β2 region of the A3G C-CDA crystal structures are observed. Importantly, these define the distortion of the β2 strand in the A3G C-CDA NMR structures and the crystal structure XRAY2-2K3A. In the XRAY1 structure with the most regular β2 geometry, residues V224, R226 and H228 are H-bonded to R238, Q237, L235 and V233, respectively (V224 interacting with both R238 and Q237). The bulged-out conformation of β2 that is observed in the XRAY2-2K3A structure coincides with H-bonding of residues V224, R226 and H228 to L235, V233 and T231, respectively. Variations on this latter arrangement are observed in each of the NMR structures, which are also characterised by a bulged out conformation of the β2 strand. Thus, multiple registers of H-bonding with the β2 of the A3G-CDA are possible and can cause this strand to be more or less structured.

### Molecular dynamics Simulations of the A3G C-CDA structures from NMR and crystallography studies

**Figure 4:**
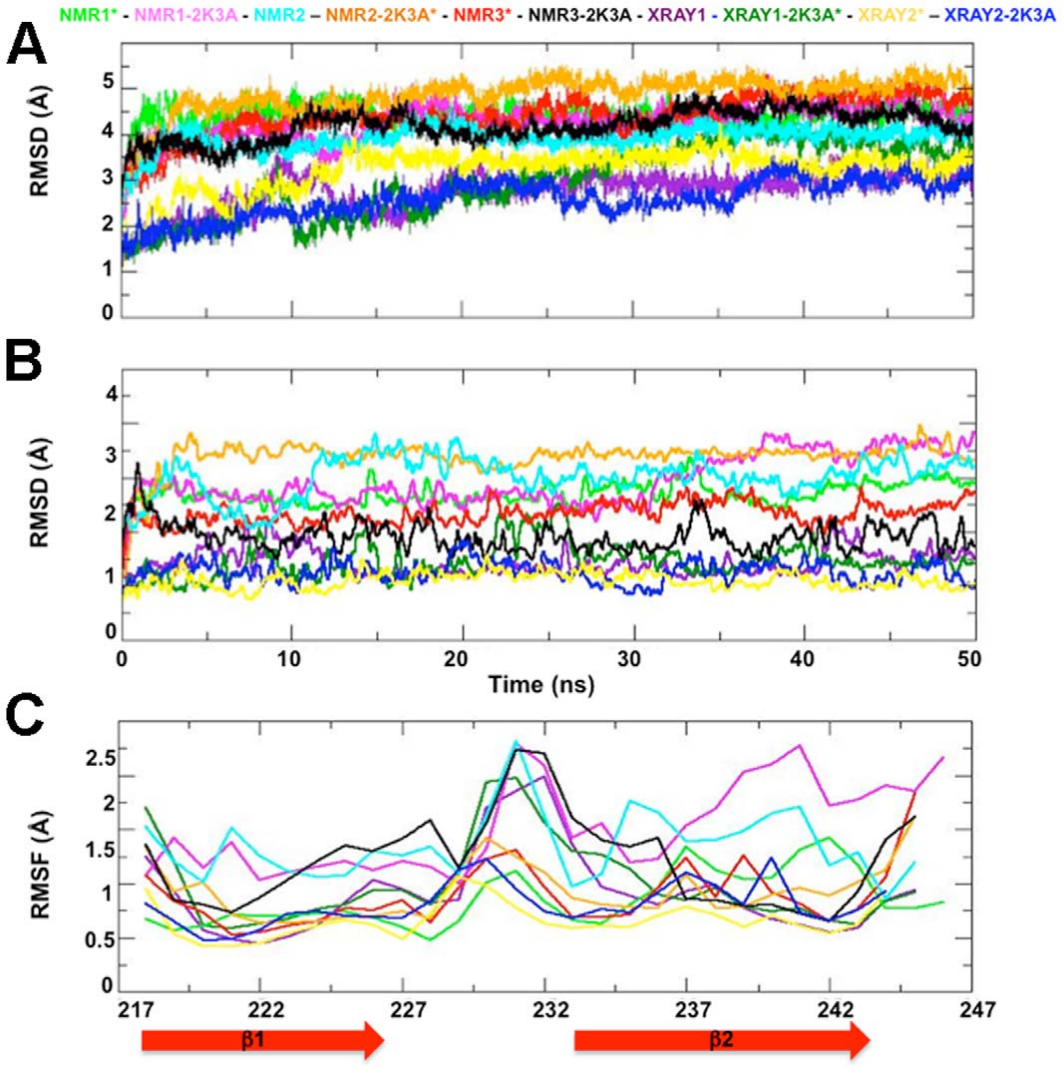
RMSD and RMSF of the A3G C-CDA Cα atoms from initial coordinates during MD simulations. (A) RMSD of the Cα atoms as function of time for the whole C-CDA domain and (B) for the β1-β2 region only. Colour code: NMR1* in light green; NMR1-2K3A in pink; NMR2 light bue; NMR2-2K3A* in orange, NMR3* in cyan; NMR3-2K3A in black; XRAY1 in purple; XRAY1-2K3A* in dark green; XRAY2* in yellow and XRAY2-2K3A dark blue. (C) RMSF of residues 217–247 with respect to their average position over the entire simulated time; the colour code is the same used (A) and (B).

We next performed 50 ns MD simulations of the A3G C-CDA high-resolution structures to assess changes from the starting conformations in a simulated aqueous environment using the GROMOS force field (see materials and methods). We performed our analyses on single monomeric C-CDA domains from the respective studies, which allowed the comparison of the structures in the absence of crystal packing interactions that may affect the protein conformations locally. To be able to compare and evaluate if the solubility-enhancing mutations from the studies by Chen (NMR1-2K3A), Harjes (NMR3-2K3A) and Shandila (XRAY2-2K3A) [28], [31], [32] affected the protein structure during the MD simulations, we introduced these mutations *in silico* into the XRAY1 and NMR2 structures to obtain the XRAY1-2K3A* and NMR2-2K3A*. Likewise, we restored the wild-type sequence in NMR1-2K3A, NMR3-2K3A and XRAY2-2K3A, obtaining the NMR1*, NMR3* and XRAY2* structures (the asterisk indicates the *in silico* generated sequences throughout). Simulations were performed in duplicate, providing a total of 20 A3G C-CDA structures for subsequent analysis. As duplicate simulations exhibited equivalent characteristics (Table S1 and Figure S3), single trajectories for each structure were randomly selected for a more detailed description. The root mean square deviation (RMSD) of the C^α^ atoms of the starting structures over the simulated time shows that all had reached equilibrium after approximately 30 ns of the simulation (Figure 4A).

Because of the differences in conformation of β2 in these structures, the RMSD of the C^α^ from the residues participating in the β1-β2 sheet (residues E217-N244) was analysed in more detail (Figure 4B). Simulations with the crystal structures XRAY1, XRAY1-2K3A*, XRAY2-2K3A and XRAY2* showed a low RMSD value of ∼1 Å, demonstrating that the starting conformation of the β2-strand remained stable during the simulations. This indicates that each of the crystal structures represent energetically stable conformations of β2. In contrast, simulations with the NMR structures showed much larger RMSD values of up to ∼ 3.5 Å for the β2 region, indicating substantial conformational changes for this part of the molecule. This is furthermore reflected in an analysis of the root mean square fluctuation (RMSF) of the residues forming the β1-β2 sheet (Figure 4C), which shows that NMR1-2K3A, NMR1* and NMR3* have particularly large fluctuations at residues N236 to F241 that make up the β2 strand. Together, these results demonstrate that a substantial rearrangement of the residues within β2 occurred during the simulations of the NMR structures, which have the least structured conformations of the β2 strand. We note that the structures with the largest RMSD in the β2 region coincide with those simulations that have the largest overall RMSD for the whole C-CDA domain (figures 4A and 4B), suggesting that rearrangement of β2 contributed to the high RMSD values observed.

### Secondary structure of the β2 strand during molecular dynamics simulations

**Figure 5:**
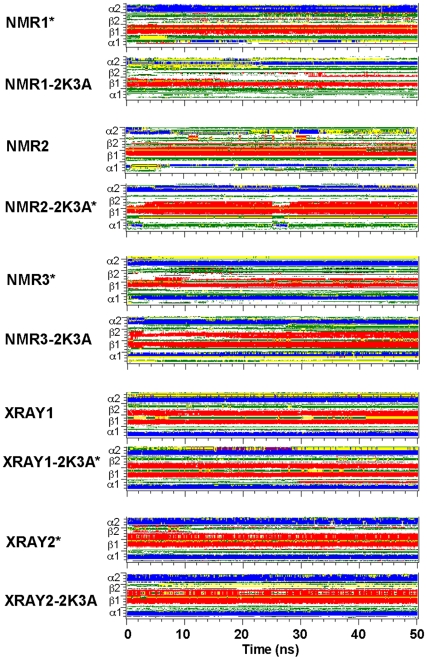
Time evolution of the secondary structure elements during MD simulations. Positions of secondary structure elements α1, β1 and β2 are indicated on the y-axis and the simulation time in nanoseconds is indicated on the x-axis. Simulations labelled with an asterisk contain *in silico* created mutations. Colours indicate secondary structure elements at a given time point as determined by DSSP classification; α-helices in blue; β-sheets in red; turns in yellow; bends in green.

To determine in more detail the conformational changes of the β2 strand, the stability of the secondary structure elements during each of the simulations was examined. We did this by plotting elements of defined secondary structure for each amino acid against the simulated time (Figure 5, Figure S3 and Figure S4). This confirmed that the starting conformation of the β2 strand from the crystal structures remained predominantly stable throughout the simulations, although temporal closing and opening of the bulge in the simulations XRAY2-2K3A and XRAY2* was observed. Simulations with the NMR structures showed a more dynamical behaviour in the sense that folding of the β2 strand was generally improved, and in some instances was periodically disrupted and reformed (Figure 5 and Figure S3). In particular, the NMR2-2K3A* and NMR3-2K3A simulations showed the most dramatic stabilization and ordering of the β2-strand, and NMR2 and NMR3* showed appreciable partial increases in formation of β2. For NMR1* and NMR1-2K3A we observed minor increases in formation of β2. These changes in the β2 region do not appear to influence other secondary structure elements (Figure S4), indicating that conformational changes leading to the formation of a more ordered β2-strand are compatible with the rest of the A3G C-CDA structure.

In order to provide a more quantitative measure for changes in secondary structure, we calculated the percentage of secondary structure content for each structure at the beginning and end of the simulation (table S1). Equivalent proportions of secondary structure were maintained during the simulations of the crystal structures. Importantly, all simulations of the NMR structures showed an increase in β-sheet content with the largest values observed for NMR2-2K3A* and NMR3-2K3A, in accordance with the results shown in Figure 4. This increase in β-strand content is mostly attributable to the regularization of β2 of these NMR structures, as β1, β3, β4 and β5 remained remarkably stable throughout the simulations (Figure S4).

### Hydrogen bonding between β1 and β2

**Figure 6:**
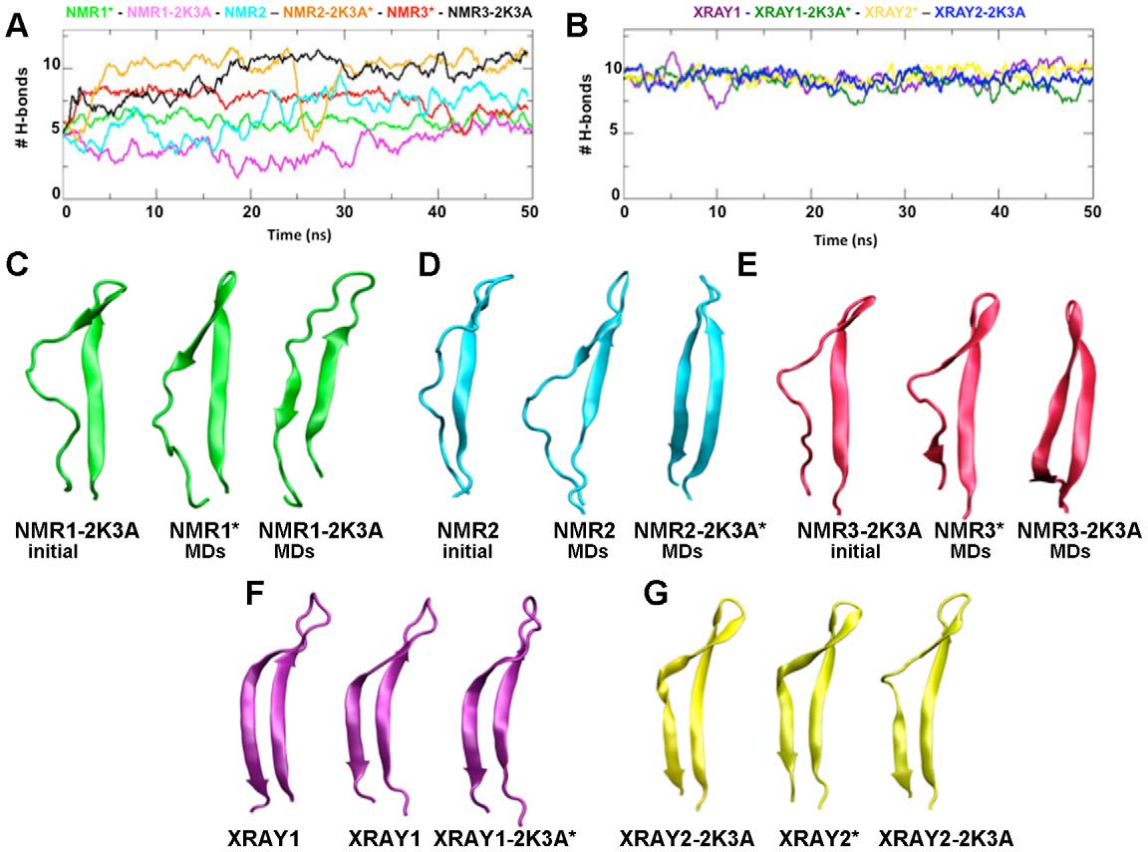
Structure of the β1-β2 sheet during MD simulations. Number of H-bonds between main-chain atoms of β1 and β2 during the simulated time. For clarity, data for simulations with NMR and crystal structures are shown in separate plots. Colour coding: (A) NMR1* in light green; NMR1-2K3A in pink; NMR2 light bue; NMR2-2K3A* in orange, NMR3* in cyan; NMR3-2K3A in black; (B) XRAY1 in purple; XRAY1-2K3A* in dark green; XRAY2* in yellow and XRAY2-2K3A dark blue. (C) Comparison of the β1-β2 sheet from the starting structures with the most representative structures derived clustering analysis of the MD simulations for NMR1-2K3A, (D) NMR2 and (E) NMR3-2K3A (F) XRAY1 and (G) XRAY2-2K3A.

Since the formation of a β-sheet is dependent on the formation of H-bonds between the two β-strands, we also analysed the number of H-bonds between the main-chain atoms of the β1-β2 sheet during the simulations (Figure 6). This analysis confirmed that XRAY1 and XRAY1-2K3A* structures maintain a β1-β2 sheet with a predominantly regular geometry and a minimum of eight H-bonds that remained stable throughout the simulation (Figure 6B). Hydrogen bonding also remained stable during the simulations of XRAY2* and XRAY2-2K3A, and during these simulations the bulged conformation of β2 was maintained. Increases in the number of β1-β2 H-bonds from the starting structure were observed with the NMR1*, NMR2, NMR2-2K3A*, NMR3* and NMR3-2K3A structures (Figure 6A). Of these, NMR1* showed the weakest stabilisation of the β1-β2 sheet by 1 to 2 H-bonds. For the NMR1-2K3A structure there was an initial loss of H-bonds during the simulation, but in the final stages this recovered to the same number of H-bonds as present in the starting structure. Interestingly, during this simulation a different part of β2 gained in definition at the expense of the defined part of the β-sheet present in the starting structure (Figure 6C). Together, these results demonstrate that the A3G C-CDA NMR structures with a poorly defined β2-strand showed a general tendency towards the formation of a more stable β1-β2 sheet and this was observed both in the absence and presence of the five solubility-enhancing mutations (Figure 6). We note, however, that the most dramatic stabilisation of β2 was observed with NMR2-2K3A* and NMR3-2K3A that both contain the solubility enhancing mutations.

We next sought to compare the networks of H-bonds in the β1-β2 sheet between the different structures. For this purpose we generated schematic representations of the β1-β2 sheet on which the most persistent H-bonds observed during the simulations are indicated (Figure S5). Comparison of the starting structures with the MD data revealed that the identity of H-bonds in the β1-β2 sheet of the crystal structures was maintained during the simulation, regardless of the presence or absence of mutations (Figure S5D and E). There was, however, a tendency to disrupt the H-bonds between T218 and N244 of the crystal structure XRAY1, which represent the closing H-bonds of the β1-β2 sheet. Formation of an H-bond between V224 and R238 was observed in the simulations with XRAY2* and XRAY2-2K3A, leading to a more closed conformation of the bulge (Figure S5E). During the simulations of the NMR structures, the H-bonding pattern was much more dynamic and, as described above, was generally characterised by an increase in interactions. This included the formation of novel H-bonds that were not present in the starting structures of either the crystal or NMR structures. Interestingly, the simulations with NMR2 and NMR3* presented stable formation of H-bonds between residues T218 to Y222 with G240 to N244, which are also present in both crystal structures (Figure S5B and C). In fact, the newly formed L220-L242 interaction from NMR2 and the T218-N244 interaction from NMR3* were among the most stable throughout the simulations.

An interesting convergence towards the H-bonding register present in the crystal structure XRAY1 was observed during the simulation with NMR3-2K3A (Figure S5C). In this case, H-bonds between residues V224, R226 and H228 were formed with Q237, L235 and V233, respectively, at the expense of the H-bonds with T231 and V233 that were present in the starting structure. This rearrangement of H-bonds coincided with regularisation of the bulge. The simulation with NMR2-2K3A* demonstrated an alternative regularisation of β2 that was achieved while maintaining the H-bonding partners of V224, R226 and H228 (Figure S5B). Thus, our analyses show that the MD simulations of the NMR structures lead to a better defined β2 strand, and that there are multiple ways in which this can be achieved.

### Hydration sites in the β1-β2 sheet

**Figure 7:**
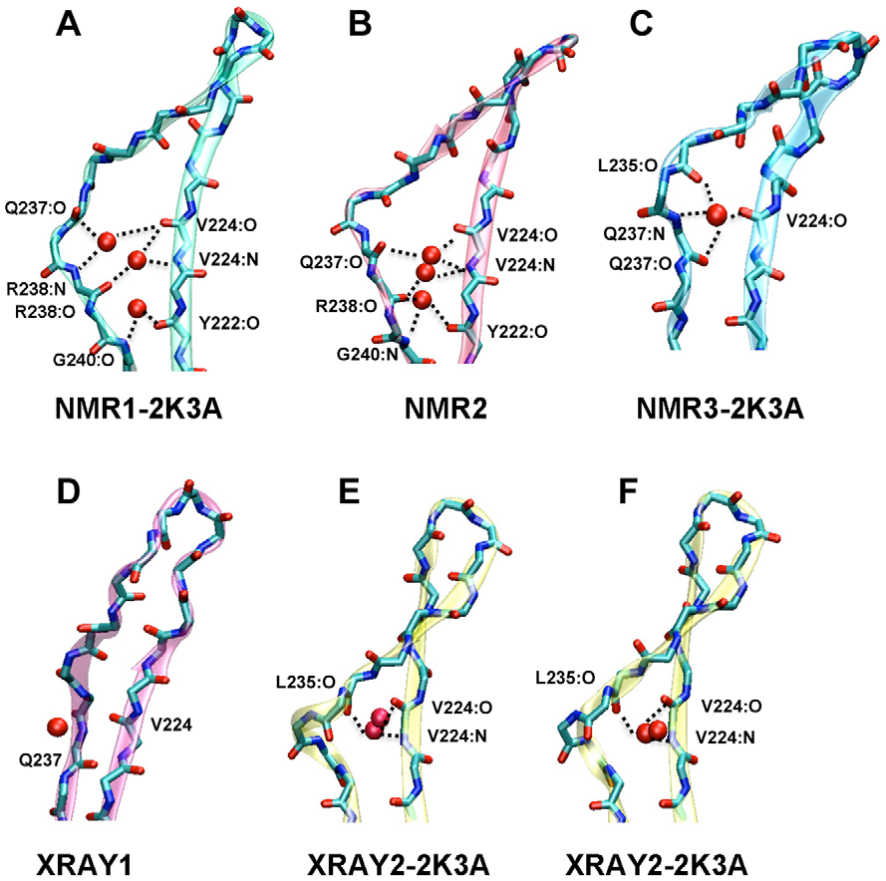
Water binding sites in the β1-β2 sheet as determined by MD solvent density analysis. Water molecules that bridge the backbone of the β1-β2 sheet are indicated in red and their interactions with amino acid residues are indicated with black dotted lines. The water-mediated interactions between the backbone of the residues V224 and Y222 in β1 and L235, Q237 and R238 in β2 are shown for: (A) NMR1-2K3A; (B) NMR2; (C) NMR3-2K3A; (D) XRAY1 and (E) XRAY2-2K3A. (F) Representation of the water molecules observed in the electron density map of the crystal structure XRAY2-2K3A.

We next set out to investigate interactions of the β1-β2 sheet with the solvent during the A3G C-CDA simulations by means of MD solvent density analysis [38]. In particular, we analysed the persistence of water molecules at the β1-β2 region by generating MD hydration maps (MDHS) for the XRAY1, XRAY2-2K3A, NMR1-2K3A, NMR2 and NMR3-2K3A structures (Figure 7). In this type of simulation, the protein structure is restrained to remain rigid, while allowing the water molecules to reach equilibrium solvation around the protein [38]. In this manner, sites within the protein structure that are particularly prone to interact with water can be identified. Because the protein structure is restrained to remain rigid, we did not include structures with *in silico* generated sequences in this analysis.

Extraction of the MDHS maps from the simulations revealed that in the NMR structures one to three water molecules are coordinated within the β1-β2 sheet by the large bulges that are present in these structures (Figure 7A, B and C). In particular, the two NMR structures with the largest bulge (NMR1-2K3A and NMR2, Figure 7A and B) both showed coordination of three water molecules by the same amino acid residues: Y222, V224 of the β1-strand and Q237, R238 and G240 of the β2-strand. In the NMR3-2K3A structure with a smaller β2 bulge, only one water molecule was observed and this bridges amino acids V224, L235 and Q237 (Figure 7C). In contrast, we did not observe preferential hydration sites between the two β-strands of the crystal structure XRAY1 with the continuous β2 strand (Figure 7D), although one water molecule from the bulk solvent remained in the proximity of V224 and Q237. Finally, the MDHS analysis performed on simulations of the XRAY2-2K3A structure predicted the presence of two persistent water molecules between residues V224 and L235 (Figure 7E). Importantly, inspection of the electron density map of the XRAY2-2K3A crystal structure revealed the presence of two water molecules at precisely this position, thereby validating our approach (Figure 7F). Together, these results demonstrate that the presence of a bulged conformation of the β2 strand is driven by hydration of residues V224, L235 and Q237, and that formation of an ordered conformation of β2 coincides with the exclusion of water molecules from the β1-β2 interface.

### Comparison of proposed DNA binding sites in the A3G C-CDA

**Figure 8:**
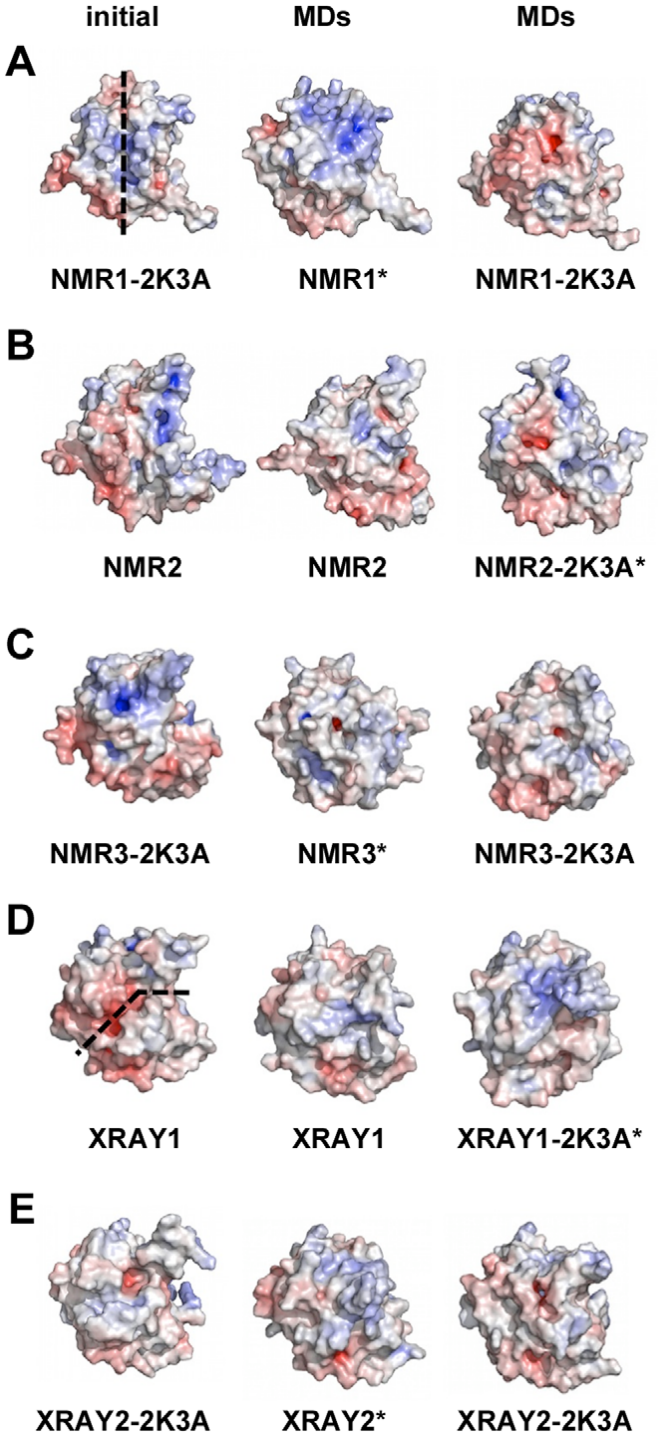
Charge distribution over the A3G C-CDA surface. Comparison of the electrostatic surface of the starting structures with the representative structures from the clustering analysis of the MD simulations. The potential is ranged from −10*κ*T (red) to +10*κ*T (blue). (A) NMR1-2K3A; (B) NMR2; (C) NMR3-2K3A; (D) XRAY1 and (E) XRAY2-2K3A. Dotted lines in (A) and (D) indicate the proposed orientation of DNA binding grooves.

We next analysed the structures of the C-CDA domain of A3G to determine whether a DNA binding site near the deaminase catalytic core could be identified. We already indicated that the positioning of putative DNA binding grooves and the identification of residues that interact with the DNA substrate differs widely between the crystallography and NMR studies. In particular, entirely different putative DNA binding grooves were proposed based on the A3G C-CDA NMR1-2K3A structure and the XRAY1 crystal structure [34](see the initial structures in Figure 8A and 8D, respectively). Curiously, the XRAY2-2K3A and NMR3-2K3A show a surface area with grooves that is similar to XRAY1, whereas NMR2 most closely resembles the groove proposed for the NMR1-2K3A structure (Figure 8). Furthermore, the charge distribution over the surface of the protein is different in each of the reported structures.

In an attempt to provide some clarity on this important subject, we sought to assess the integrity of the proposed DNA binding grooves and the charge distribution over the protein surface during the MD simulations. For this purpose, we performed a clustering analysis on each of the MD trajectories to identify the most populated cluster from which a representative structure could be derived to compare with the starting structure. Upon selection of this representative structure, the electrostatic charge distribution was analysed (Figure 8). We also calculated the solvent accessible surface areas (SASA) of the protein surface for each of the initial and representative MD structures to highlight the presence of grooves or pockets (Figure S6). Together, these sets of data demonstrate that (1) the putative DNA binding grooves from the XRAY1, XRAY2-2K3A and NMR1-2K3A structures were not maintained during the MD simulations, and (2) a wide variety of possible different pockets or grooves on the surface of the A3G C-CDA, as well as a diverse charge distribution, were displayed throughout the MD simulations.

**Figure 9:**
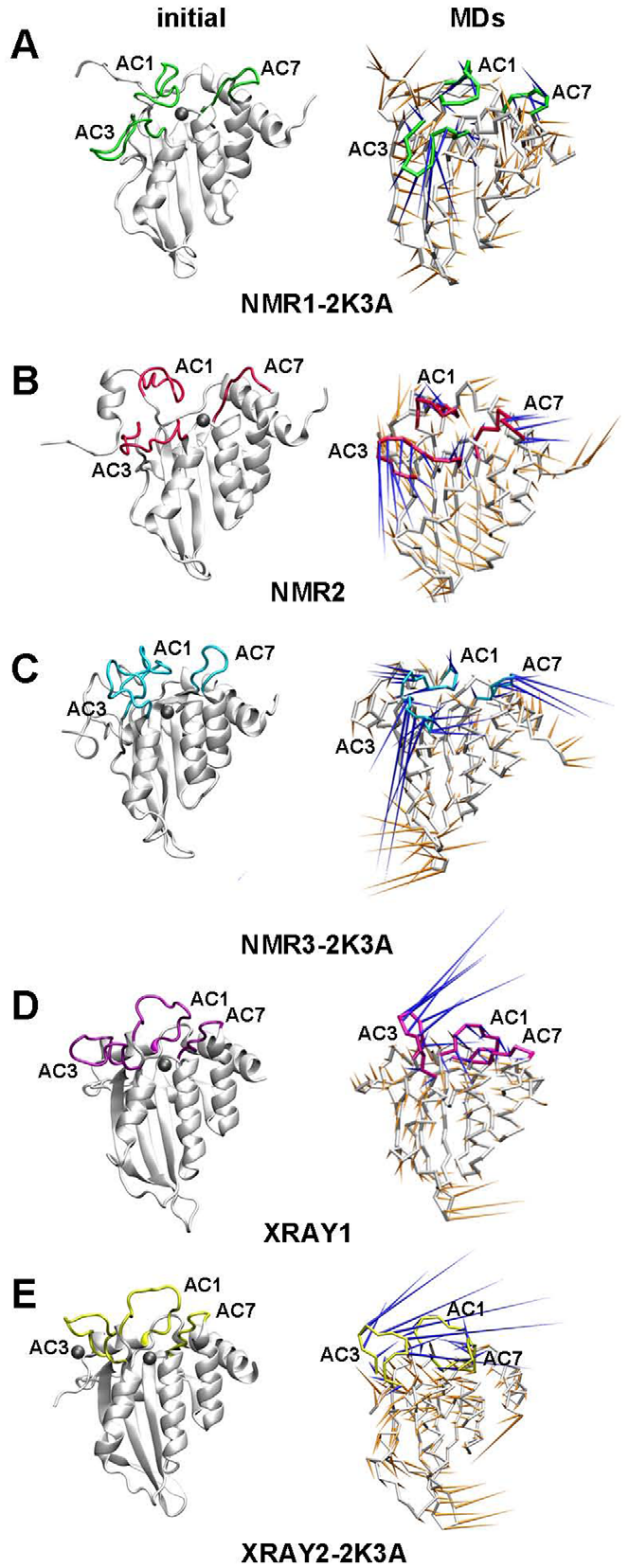
Conformation of loops near the CDA catalytic core and their dynamics during the simulations. Ribbon representations of the A3G C-CDA starting structures (left had column) and structures after PCA analysis (right hand column) with loops near the catalytic core highlighted in colour: (A) NMR1-2K3A in green; (B) NMR2 in magenta; (C) NMR3-2K3A in blue; (D) XRAY1 in pink and (E) XRAY2-2K3A in yellow. Each Cα atom has a cone attached pointing in the direction of the motion described for the first eigenvector for that atom, with the size of the cone proportional to the amplitude of motion. Cones for residues in loops near the catalytic core are shown in blue; all others are shown in orange. Zinc ions are indicated by grey spheres. In the XRAY2-2K3A structure a second zinc ion that is remote from the catalytic core was observed. Plots for simulations with *in silico*-generated sequences are not shown.

To investigate in some detail the source of these differences, we generated ribbon models of the C-CDA on which residues R215, E259 and D316 are indicated (Figure S7). These represent the three amino acids that were commonly identified as mediating interactions with the DNA substrate by three different studies [28]–[30]. This representation of the intitial and representative MD structures highlights that there is considerable variability in the positioning of loops and side chains, which underlies the aforementioned divergence in exposed surface area and charge distribution. Indeed, the variable positioning of loops AC1, AC3 and AC7 that largely determine the accessibility of the catalytic core is evident from a comparison of the starting structures from the crystallography and NMR studies (Figure 9). These loops consistently emerged as being the most flexible parts of the A3G C-CDA throughout our set of MD simulations, as identified by a principal component analysis (PCA) (Figure 9). The considerable flexibility of these loops will also have contributed to the relatively high RMSD values of the structures during the simulations (Figure 4A). As we did not observe the formation of a common stable conformation of loops near the catalytic core during MD simulations, we conclude that there is no evidence to support the structure of any of the previously proposed DNA binding sites within the A3G C-CDA domain. This would suggest that DNA binding to the C-CDA of A3G may instead occur by an induced fit mechanism. The interaction of A3G with DNA is known to be dynamic and most likely short-lived as there is ample evidence to support that A3G can translocate along the DNA to edit multiple target sites on a single DNA substrate [24], [39], [40]. This dynamical behaviour may also underlie the current absence of high resolution structures of A3G bound to DNA.

## Discussion

We have performed a MD study of high-resolution structures of the A3G C-CDA domain that was prompted by considerable differences in the integrity of the β2 strand as well as the organisation and location of a putative nucleic acid binding site. As three of these structures contained five solubility enhancing mutations, simulations were performed with and without these mutations. We confirmed empirically that A3G maintains DNA editing and virus inhibition activities in the presence of these mutations.

The β2 strand appears relatively ordered in the crystal structure XRAY1 but adopts a variety of bulged out conformations in the NMR structures as well as the second crystal structure XRAY2-2K3A. Simulations with the crystal structures demonstrated that the starting conformation of the β2 strand remained stable, regardless of the presence or absence a bulge interrupting the strand. On the other hand, a common behaviour for simulations with NMR structures was observed in the spontaneous formation of a better ordered and stable β2 strand, as measured in terms of secondary structure and number of H-bonds involved in the β1-β2 sheet (Figures 5 and 6). We observed a wide range of possible H-bonding registers within the β1-β2 sheet, which was not limited to the H-bonds present in the starting structures. Indeed, stabilisation of the β2 strand was achieved by a variety of H-bonding patterns.

The β2 strand was not universally stabilised in our set of simulations, and we demonstrated that this is due to differences in the hydration of residues in the β1-β2 sheet. The presence of a bulged β2 seems due to a particularly stable hydration site around residues Y222, V224 of β1 and L235, Q237 and R238 of β2. In the NMR structures with a bulged β2 strand, these residues coordinate a different number of water molecules that is dependent on the extent of the bulge. For instance, the NMR1-2K3A structure has the most severely disordered β2 strand that can coordinate three water molecules and showed the least stabilisation of β2 during the simulations. On the other hand, simulations with the XRAY1 structure in which the β2 strand adopts an ordered conformation, we observed that water molecules were excluded from the β1-β2 sheet.

The conformation of the β2 strand in the A3G C-CDA is of particular interest because an ordered β2 would allow the juxtaposition of β-sheets in the N- and C-CDAs of A3G as is observed in the multimeric crystal structure of the closely related A2 protein [33]. The formation of an extended β-sheet through β2-β2 interactions in APOBEC proteins appears to be an evolutionary conserved feature of these proteins as, in addition to the A2 crystal structure, there is evidence to suggest that the AID and A3C proteins are assembled in a similar manner [33], [41]. In this regard, it was surprising that several of the A3G C-CDA structures contained a distorted conformation of β2 that would disallow the assembly of an elongated β-sheet consisting of the N- and C-CDA domains. As all available structures of the A3G C-CDA to date were obtained with constructs from which the N-CDA was deleted, an exposed β2 strand at the edge of the structure may not necessarily reflect its presentation or integrity within the full length molecule. Indeed, it is possible that this may have been the cause of the different conformations of β2 observed in the various structures.

The presence of a predominantly ordered β2 in the XRAY1 crystal structure, as well as the improved definition of β2 during simulations of the NMR structures, would be consistent with the assembly of the N- and C-CDA domains into an elongated β-sheet. However, we recognise that the minimal distortion of β2 as observed in the crystal structure XRAY1 would still introduce some deviation from the neatly ordered sheet that is observed in the structure of A2. This would imply that if the folding of the full-length A3G protein does proceed by β2-β2 interactions, the resulting β-sheet may not be as ordered as was previously predicted based on homology modelling using A2 as a template [12], [16], [35](Figure S1). Alternatively, given the diverse array of H-bond networks within the β1-β2 sheet observed in the starting structures and during the simulations, it remains possible that the proximity of the β2 from the N-CDA may induce a specific H-bonding register. In other words, assembly of the N- and C-CDA domains via β2-β2 interactions could favour one of the more ordered conformations of β2, such as those observed in simulations with NMR2-2K3A* or NMR3-2K3A, over the bulged conformation. This would also be consistent with our observation that the bulge is stabilised by bound water molecules, rather than a stable conformation of the protein backbone itself.

It has been suggested that assembly of a full-length A3G model through β2-β2 interactions would additionally be prevented by the inability to link the α6 of the N-CDA to the α1 of the C-CDA through the residues E191-P199, based on the positioning of α1 in the NMR3-2K3A structure [31]. In general, definition of α1 in the NMR studies has not been as good as in the two crystal structures, which both show that α1 is closely packed against the β-sheet of the A3G C-CDA (Figure 1) and in a position that would not restrict the linkage with α6 of the N-CDA (not shown). Together with our analyses of the structure of the A3G C-CDA domain and its interactions with the solvent during the molecular dynamics trajectories this indicates that the possibility of folding the full-length A3G protein via β2-β2 interactions should not be ruled out.

A definitive positioning of a single stranded DNA binding site within the A3G C-CDA has also been much debated [28], [29], [34]. Our analysis of the exposed surfaces for the resolved structures and the MD simulations thereof further demonstrated the ambiguity in attempting to identify a pre-defined DNA binding site within the A3G C-CDA. In addition, we demonstrated that in the absence of a bound DNA substrate the charged residues involved in the DNA binding are distributed dynamically over the protein surface. We showed that differences in positioning of loops AC1, AC3 and AC7 at present preclude the assignment of a clearly defined DNA binding pocket within the A3G C-CDA. Loops AC1, AC3 and AC7 are all in close proximity to the deaminase catalytic core and contain many of the residues that are thought to contribute to DNA substrate binding [28]–[30], [42], [43]. In particular, these loops proved the most flexible regions of the molecule during the simulations (Figure 9). We note that the conformational ambiguity of β2 may have contributed to confounding the identification of a DNA binding site. In the primary sequence of the A3G C-CDA, β2 is directly followed by loop AC3 and the nature of H-bonding between β1 and β2 would thus affect the size and orientation of this loop.

Binding of DNA at the C-CDA of A3G is known to be influenced by the N-CDA. In particular, the inclusion of this domain imparts a higher affinity for the DNA substrate to A3G than the isolated C-CDA by lowering the dissociation constant from approximately 400 µM to approximately 50 nM [23], [24], [28]. In addition, the 3′ to 5′ directional bias of deamination is only observed upon inclusion of the N-CDA [15]. It is currently unknown whether this is due to direct contribution of the N-CDA to DNA binding or indirectly through structural effects that may determine the conformations of β2, AC1, AC3 and AC7 of the C-CDA. The former may potentially be reflected in the inhibitory effect of RNA binding to A3G on DNA editing [23], [39,39], as the association of A3G with RNA has consistently been attributed to the N-CDA domain [12], [14], [16], [44]. Thus, fully resolving the controversy surrounding the interaction of A3G with its substrate DNA is likely to depend on the inclusion of the N-CDA. In the absence of such data, our current MD analyses point to considerable flexibility of loops in the proximity of the catalytic core, which is most readily reconciled with an induced-fit mechanism for the binding of single stranded DNA substrate to the A3G C-CDA.

In this study, we have set out to investigate the differences between various high-resolution structures of the A3G C-CDA domain, which are primarily defined by differences in conformation of the β2 strand and access to the catalytic core. Importantly, we have shown that ordered conformations of β2 emerged during MD simulations of the NMR structures that had a disordered starting conformation of β2. An ordered β2 in the A3G C-CDA would in turn allow the assembly of the N- and C-CDA domains into an elongated β-sheet, as is observed in the closely related A2 protein. We have furthermore shown that conflicting reports concerning the identification of a DNA binding site within the A3G C-CDA are due to differential positioning and the inherent flexibility of loops near the deaminase catalytic core. Thus, our analyses have provided some insight into these much debated facets of A3G and may inform the unravelling of the interactions of A3G with its nucleic acid substrates. For example, the precise mechanism behind the differential target site recognition of different A3 proteins on DNA substrates and the identity of specific RNAs that bind to A3 proteins remain poorly understood. In the absence of an A3G structure bound to substrate, or indeed the structure of the full-length protein, modelling efforts such as those presented here provide an alternative method for further addressing these issues.

## Materials and Methods

### Molecular Dynamics Simulations

MD simulations were performed with the following structures from the PDB: 2JYW, 2KBO, 2KEM, 3E1U and 3IR2. The nomenclature adopted here as well as the original references are given in table 1. For simulations of structures derived from NMR studies, the top ranked structure from the deposited bundle, representing the lowest energy structure, was selected. We also performed MD simulations of mutant version of these structures, which were generated *in silico* using *PyMOL* (www.pymol.org). Calculations were performed with the GROMACS package, [45] using the GROMOS96 force field [46]. Simulations were performed at pH = 7 and the protonation states of pH-sensitive residues were as follows: Arg and Lys were positively charged, Asp and Glu were negatively charged, and His was neutral. The net charge of the protein was neutralized by the addition of Cl^−^ and Na^+^ ions. The systems were solvated in a box of 80 Å×80 Å×80 Å and SPC water molecules (approximately 16000) were added [47], in a solution of 50 mM NaCl. Periodic boundary conditions were applied and the Berendsen's algorithm [48] for temperature and pressure coupling was adopted (300 K and 1 atm, respectively). After a first steepest descent energy minimization with positional restraints on the solute, the LINCS algorithm was used to constrain the bonds [49] and to carry out an initial 200 ps simulation with the positions of the solute atoms restrained by a force constant of 3000 kJ/(mol nm^2^) to let the water diffuse around the molecule and for equilibration. The particle mesh Ewald method (PME) [50] was used for the calculation of electrostatic contribution to non bonded interactions (grid spacing of 0.12 nm) with a cut-off of 1.4 nm and a time step of 2 fs. The GROMACS package and self-written programs have been used for the analysis of the data. SASA values were calculated with the POPS program [51].The MD solvent distribution was calculated as described previously [38]. The Dynamite Server (www.biop.ox.ac.uk) was used to produce PCA analysis of the MD trajectories. Secondary structure analysis was performed with DSSP [52]. Images were generated with visual molecular dynamics (VMD 1.8.5.) [53].

### Plasmids and cloning

Wild type and mutant A3G expression plasmids for infectivity studies and the bacterial editing assay were generated as described previously [12], [54].

### Single-cycle infectivity assays

Stocks of HIV-1/Δ*vif* [55] were prepared by cotransfection of 35-mm diameter monolayers of 293T cells with 0.5 µg of pA3G expression vector and 1.0 µg of pIIIB/Δ*vif* using polyethylenimine (PEI). After 24 hr, the supernatants were harvested and volumes corresponding to 5 ng p24^Gag^ used to infect 10^5^ TZM-bl indicator cells. The producer cells were lysed in SDS-containing loading dye for the analysis of protein expression. The induced expression of β-galactosidase in whole cell lysates was measured 24 hr after the initiation of infection using the Galacto-Star system (Applied Biosystems).

### Analysis of protein expression by immunoblotting

Whole cell lysates prepared from virus producing cells were resolved by SDS-polyacrylamide gel electrophoresis (SDS-PAGE, 11% gel) and analysed by immunoblotting using primary antibodies specific for A3G [18] and Hsp90 (sc7947: Santa Cruz). Blots were resolved using fluorescent secondary antibodies and the LI-COR infrared imaging technology (LI-COR UK LTD).

### *E. coli* mutation assay

The KL16 strain of *E. coli* was transformed with pTrc99A-based, IPTG-inducible A3G expression vectors or the empty vector [37]. Individual colonies were picked and grown to saturation in LB medium containing 100 µg/ml ampicillin and 1 mM IPTG. Appropriate dilutions were spread onto agar plates containing either 100 µg/ml ampicillin or 100 µg/ml rifampicin and incubated overnight at 37°C. Mutation frequencies were recorded as the number of rifampicin-resistant colonies per 10^9^ viable cells, which were enumerated using the ampicillin-containing plates. Colony counts were recorded in this manner on 12 rifampicin- and 12 ampicilin-containing plates for each construct, in sets of 4 of each at one time. To average the repeat experiments, the average colony count for wild type A3G was set at 100 and all other scores were normalized to this value.

## Supporting Information

Figure S1 Implications of the conformation of β2 for the folding of full-length A3G. View of the extended β-sheet that connects the N- and C-CDA in a homology model of full-length A3G as a dimer [12]. Monomer subunits are shown in magenta and green. The inner N-CDA domains mediate dimerisation of A3G and the catalytically active C-CDA domains are on the outer part of the model. In the left-hand monomer subunit of the full-length A3G homology model (shown in magenta), the structure of the XRAY1 C-CDA is superimposed (shown in blue). (B) Close up of the proposed β2-β2 interaction in the model of full-length A3G showing the β1-β2 sheets from the N- and C-CDA in magenta. (C) The distorted β2 sheet observed in NMR-2K3A is shown in blue and impedes interaction with β2 of N-CDA model. (D) The ordered conformation of β2 observed in XRAY1 is shown in blue and would be consistent with connecting the N- and C-CDA domains through β2-β2 interactions.
(1.67 MB PDF)

Figure S2 Positions of solubility enhancing mutations in A3G-2K3A. (A) A ribbon model of the NMR1-2K3A structure (PDB code 2JYW) is shown with the positions of the five solubility enhancing mutations shown in magenta. The same structure is shown in (B) after rotation by 180°.
(0.40 MB PDF)

Figure S3 Time evolution of the β1-β2 sheet during duplicate MD simulations. Positions of secondary structure elements α1, β1, β2 and α2 are indicated on the y-axis and the simulation time in nanoseconds is indicated on the x-axis. Simulations labelled with an asterisk contain *in silico* created mutations. Colours indicate secondary structure elements at a given time point as determined by DSSP classification; α-helices in blue; β-sheets in red; turns in yellow; bends in green. Duplicate simulations are indicated as MD1 and MD2. Simulations described in detail in the text correspond to the data from MD1.
(6.49 MB PDF)

Figure S4 Time evolution of the secondary structure elements during MD simulations. Positions of secondary structure elements α-helices 1 through 6 and, β-strands 1 through 5 are indicated on the y-axis and the simulation time in nanoseconds is indicated on the x-axis. Simulations labelled with an asterisk contain *in silico* created mutations. Colour indicate secondary structure elements at a given time point as determined by DSSP classification; α-helices in blue; β-sheets in red; turns in yellow; bends in green.
(4.07 MB PDF)

Figure S5 H-bonding between β1 and β2 in A3G initial structures and during simulations. Schematic representations of the β1-β2 sheet with H-bonds between the main-chain atoms indicated by dotted lines. H-bonds present in the initial structures are indicated in black. H-bonds observed during the simulations are colour coded to indicate the life time as a percentage of the total simulation time: 20%–60% in green, 61% to 80% in blue and 81% to 100% in red. The left column shows the β1–β2 sheet for the initial structures, the middle column for simulations with the wild-type sequence and the right column for simulations with the 2K3A mutations. Mutated residues are indicated in red. (A) NMR1-2K3A, (B) NMR2, (C) NMR3-2K3A, (D) XRAY1 and (E) XRAY2-2K3A.
(1.21 MB PDF)

Figure S6 Exposed surface area of the A3G C-CDA. Comparison of the exposed surface area of starting structures with the representative structures from the clustering analysis of MD simulations. Residues indicated in purple have a SASA value greater than 90 Å^2^ and those indicated in green have a SASA value lower than 40 Å^2^. SASA values were calculated with the POPS program. (A) NMR1-2K3A; (B) NMR2; (C) NMR3-2K3A; (D) XRAY1 and (E) XRAY2-2K3A.
(4.89 MB PDF)

Figure S7 Positioning of amino acids that mediate interactions of the A3G C-CDA with the DNA substrate. Comparison starting structures with the most representative structure extracted by clustering analysis from the MD simulations as ribbon representations. Amino acid residues R215, E259 and D316 are shown in stick representations and are indicated with the letters R, E and D, respectively. These three amino acids represent the agreement between three independent studies reporting residues within the A3G C-CDA that mediated interactions with the DNA substrate [28]–[30]. The zinc ion at each catalytic core is shown as a grey sphere. (A) NMR1-2K3A; (B) NMR2; (C) NMR3-2K3A; (D) XRAY1 and (E) XRAY2-2K3A.
(3.63 MB PDF)

Table S1 Percentage of secondary structure in the A3G C-CDA domain before and after simulations. Secondary structure was calculated with the DSSP algorithm for the initial and representative structures from clustering analysis. We performed duplicate simulations with each structure, which are marked as MD1 and MD2 in the table. Simulations described in detail in the text correspond to the data from MD1.
(0.15 MB DOC)
