# Supplementary material for: Rationalisation of the Differences between APOBEC3G Structures from Crystallography and NMR Studies by Molecular Dynamics Simulations
Source: PLoS One. 2010 Jul 12;5(7):e11515. doi: 10.1371/journal.pone.0011515 (PMC2902501; doi:10.1371/journal.pone.0011515)
Supplement: Figure S1 — Implications of the conformation of β2 for the folding of full-length A3G. View of the extended β-sheet that connects the N- and C-CDA in a homology model of full-length A3G as a dimer [12]. Monomer subunits are shown in magenta and green. The inner N-CDA domains mediate dimerisation of A3G and the catalytically active C-CDA domains are on the outer part of the model. In the left-hand monomer subunit of the full-length A3G homology model (shown in magenta), the structure of the XRAY1 C-CDA is superimposed (shown in blue). (B) Close up of the proposed β2-β2 interaction in the model of full-length A3G showing the β1-β2 sheets from the N- and C-CDA in magenta. (C) The distorted β2 sheet observed in NMR-2K3A is shown in blue and impedes interaction with β2 of N-CDA model. (D) The ordered conformation of β2 observed in XRAY1 is shown in blue and would be consistent with connecting the N- and C-CDA domains through β2-β2 interactions. (1.67 MB PDF) [file pone.0011515.s001.pdf]

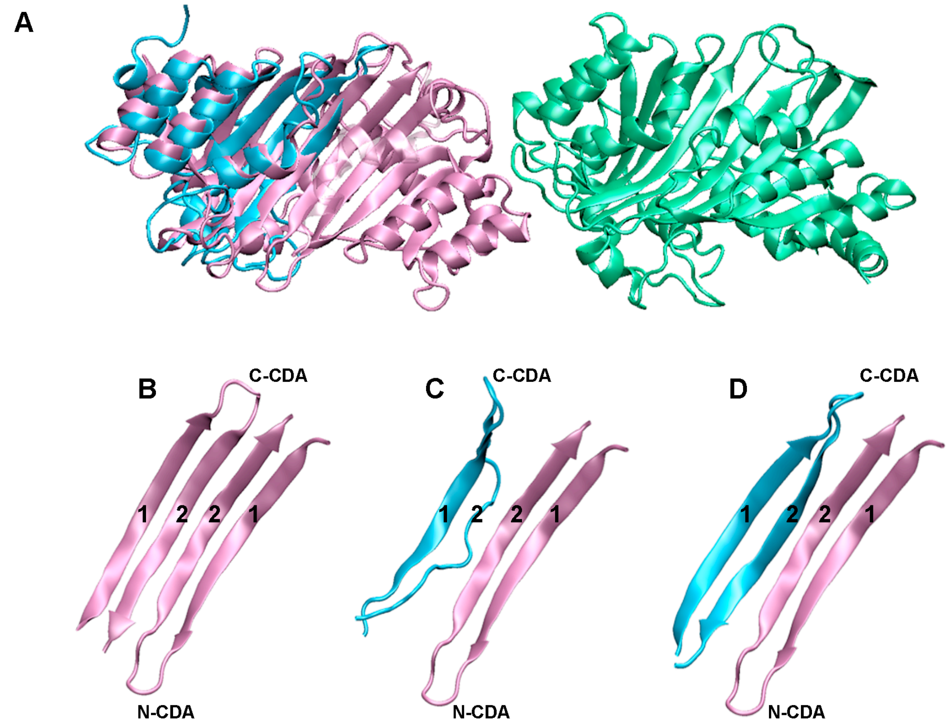

Figure S1. Implications of the conformation of β2 for the folding of full-length A3G. (A) View of the extended β-sheet that connects the N- and C-CDA in a homology model of full-length A3G as a dimer [12]. Monomer subunits are shown in magenta and green. The inner N-CDA domains mediate dimerisation of A3G and the catalytically active C-CDA domains are on the outer part of the model. In the left-hand monomer subunit of the full-length A3G homology model (shown in magenta), the structure of the XRAY1 C-CDA is superimposed (shown in blue) (B) Close up of the proposed β2-β2 interaction in the model of full-length A3G showing the β1-β2 sheets from the N- and C-CDA in magenta. (C) The distorted β2 strand observed in NMR-2K3A is shown in blue and impedes interaction with β2 of N-CDA model. (D) The ordered conformation of β2 observed in XRAY1 is shown in blue and would be consistent with connecting the N- and C-CDA domains through β2-β2 interactions.
