# Supplementary material for: Rationalisation of the Differences between APOBEC3G Structures from Crystallography and NMR Studies by Molecular Dynamics Simulations
Source: PLoS One. 2010 Jul 12;5(7):e11515. doi: 10.1371/journal.pone.0011515 (PMC2902501; doi:10.1371/journal.pone.0011515)
Supplement: Figure S2 — Positions of solubility enhancing mutations in A3G-2K3A. (A) A ribbon model of the NMR1-2K3A structure (PDB code 2JYW) is shown with the positions of the five solubility enhancing mutations shown in magenta. The same structure is shown in (B) after rotation by 180°. (0.40 MB PDF) [file pone.0011515.s002.pdf]

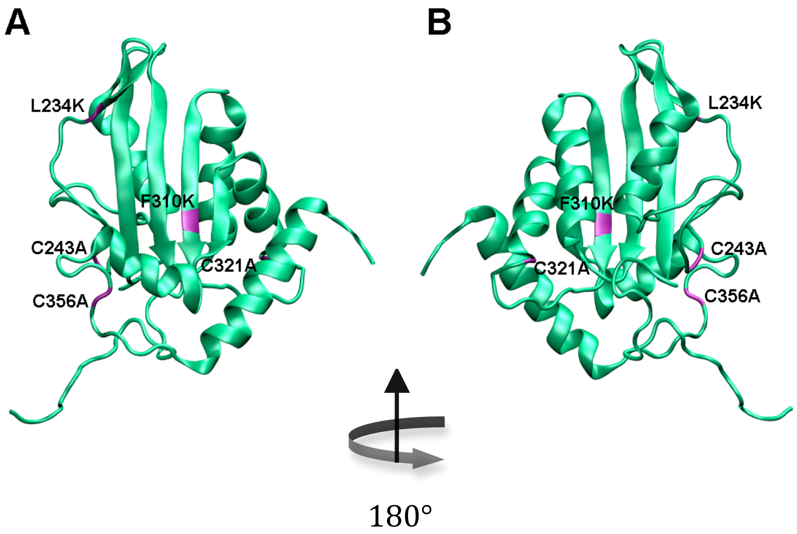


**Figure S2. Positions of solubility enhancing mutations in A3G-2K3A.** (A) A ribbon model of the NMR1-2K3A structure (PDB code 2JYW) is shown with the positions of the five solubility enhancing mutations shown in magenta. The same structure is shown in (B) after rotation by 180°.
