# Supplementary material for: Rationalisation of the Differences between APOBEC3G Structures from Crystallography and NMR Studies by Molecular Dynamics Simulations
Source: PLoS One. 2010 Jul 12;5(7):e11515. doi: 10.1371/journal.pone.0011515 (PMC2902501; doi:10.1371/journal.pone.0011515)
Supplement: Figure S3 — Time evolution of the β1-β2 sheet during duplicate MD simulations. Positions of secondary structure elements α1, β1, β2 and α2 are indicated on the y-axis and the simulation time in nanoseconds is indicated on the x-axis. Simulations labelled with an asterisk contain in silico created mutations. Colours indicate secondary structure elements at a given time point as determined by DSSP classification; α-helices in blue; β-sheets in red; turns in yellow; bends in green. Duplicate simulations are indicated as MD1 and MD2. Simulations described in detail in the text correspond to the data from MD1. (6.49 MB PDF) [file pone.0011515.s003.pdf]

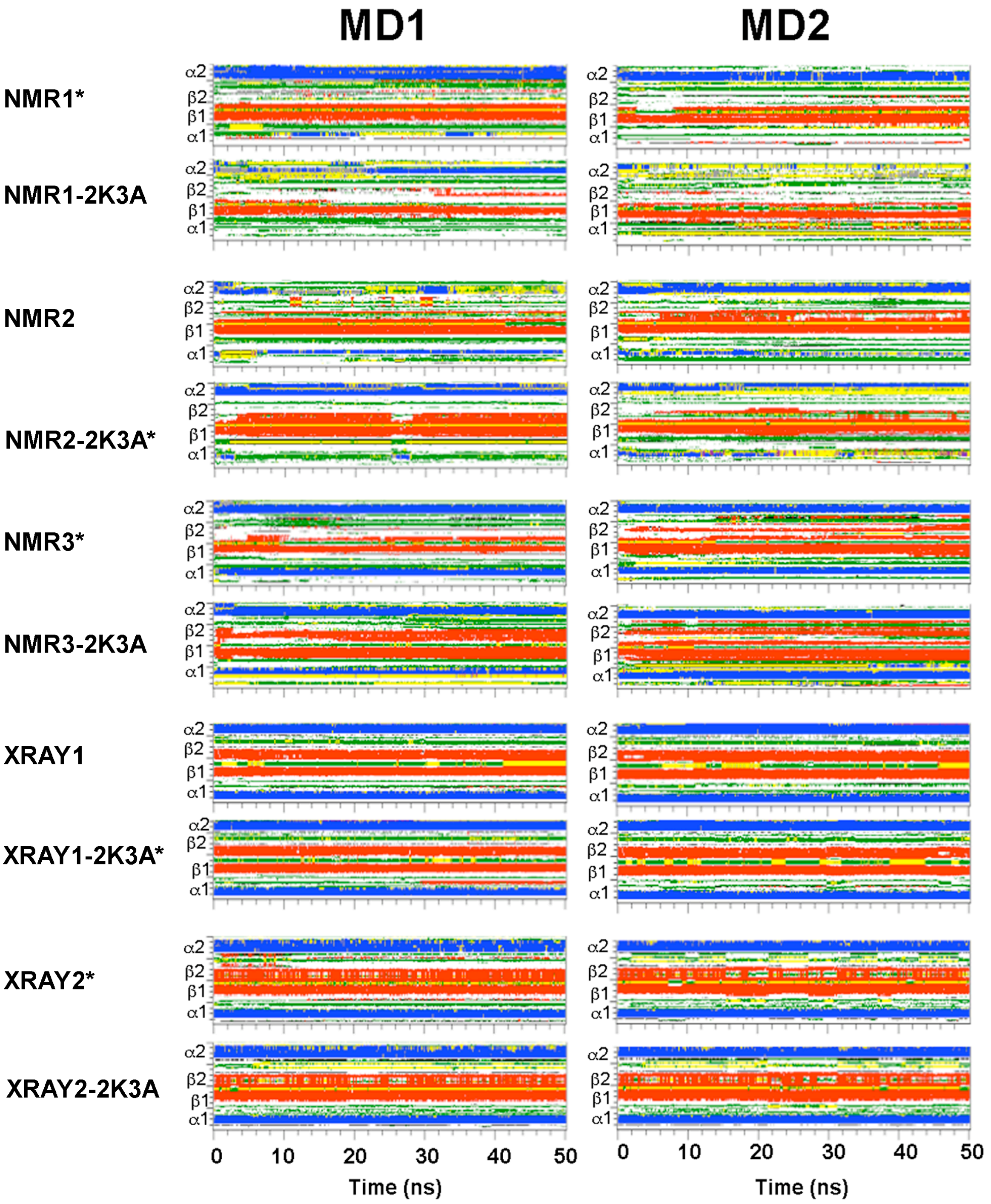

Figure S3. Time evolution of the β1- β2 sheet during duplicate MD simulations. Positions of secondary structure elements α1, β1, β2 and α2 are indicated on the y-axis and the simulation time in nanoseconds is indicated on the x-axis. Simulations labelled with an asterisk contain *in silico* created mutations. Colours indicate secondary structure elements at a given time point as determined by DSSP classification; α-helices in blue; β-sheets in red; turns in yellow; bends in green. Duplicate simulations are indicated as MD1 and MD2. Simulations described in detail in the text correspond to the data from MD1.
