# Supplementary material for: Rationalisation of the Differences between APOBEC3G Structures from Crystallography and NMR Studies by Molecular Dynamics Simulations
Source: PLoS One. 2010 Jul 12;5(7):e11515. doi: 10.1371/journal.pone.0011515 (PMC2902501; doi:10.1371/journal.pone.0011515)
Supplement: Figure S5 — H-bonding between β1 and β2 in A3G initial structures and during simulations. Schematic representations of the β1-β2 sheet with H-bonds between the main-chain atoms indicated by dotted lines. H-bonds present in the initial structures are indicated in black. H-bonds observed during the simulations are colour coded to indicate the life time as a percentage of the total simulation time: 20%–60% in green, 61% to 80% in blue and 81% to 100% in red. The left column shows the β1–β2 sheet for the initial structures, the middle column for simulations with the wild-type sequence and the right column for simulations with the 2K3A mutations. Mutated residues are indicated in red. (A) NMR1-2K3A, (B) NMR2, (C) NMR3-2K3A, (D) XRAY1 and (E) XRAY2-2K3A. (1.21 MB PDF) [file pone.0011515.s005.pdf]

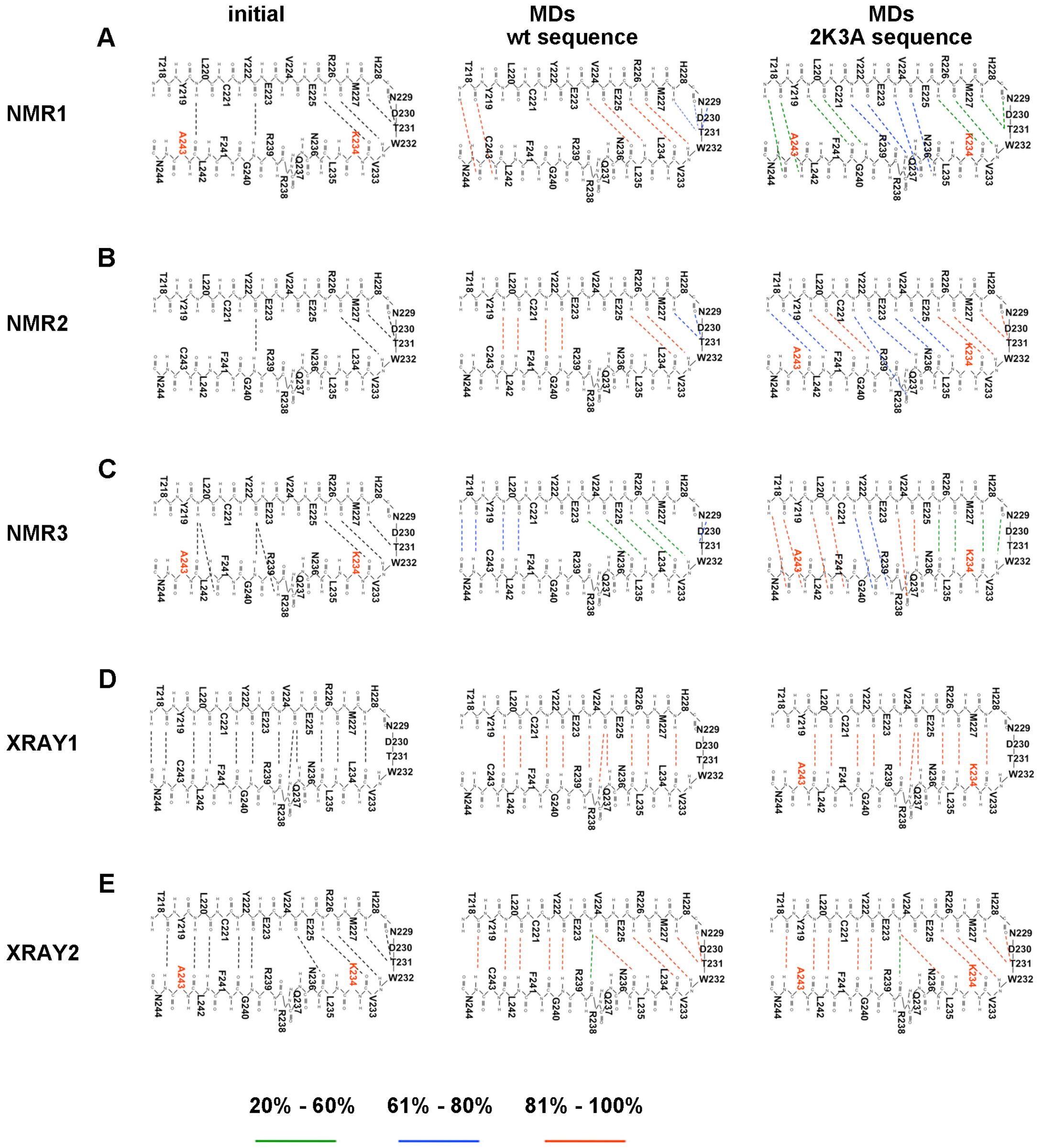


**Figure S5. H-bonding between β1 and β2 in A3G intitial structures and during simulations.** Schematic representations of the β1-β2 sheet with H-bonds between the main-chain atoms indicated by dotted lines. H-bonds present in the initial structures are indicated in black. H-bonds observed during the simulations are colour coded to indicate the life time as a percentage of the total simulation time: 20%-60% in green, 61% to 80% in blue and 81% to 100% in red. The left column shows the β1-β2 sheet for the initial structures, the middle column for simulations with the wild-type sequence and the right column for simulations with the 2K3A mutations. Mutated residues are indicated in red. (A) NMR1-2K3A, (B) NMR2, (C) NMR3-2K3A, (D) XRAY1 and (E) XRAY2-2K3A.
