# Supplementary material for: Rationalisation of the Differences between APOBEC3G Structures from Crystallography and NMR Studies by Molecular Dynamics Simulations
Source: PLoS One. 2010 Jul 12;5(7):e11515. doi: 10.1371/journal.pone.0011515 (PMC2902501; doi:10.1371/journal.pone.0011515)
Supplement: Figure S6 — Exposed surface area of the A3G C-CDA. Comparison of the exposed surface area of starting structures with the representative structures from the clustering analysis of MD simulations. Residues indicated in purple have a SASA value greater than 90 Å2 and those indicated in green have a SASA value lower than 40 Å2. SASA values were calculated with the POPS program. (A) NMR1-2K3A; (B) NMR2; (C) NMR3-2K3A; (D) XRAY1 and (E) XRAY2-2K3A. (4.89 MB PDF) [file pone.0011515.s006.pdf]

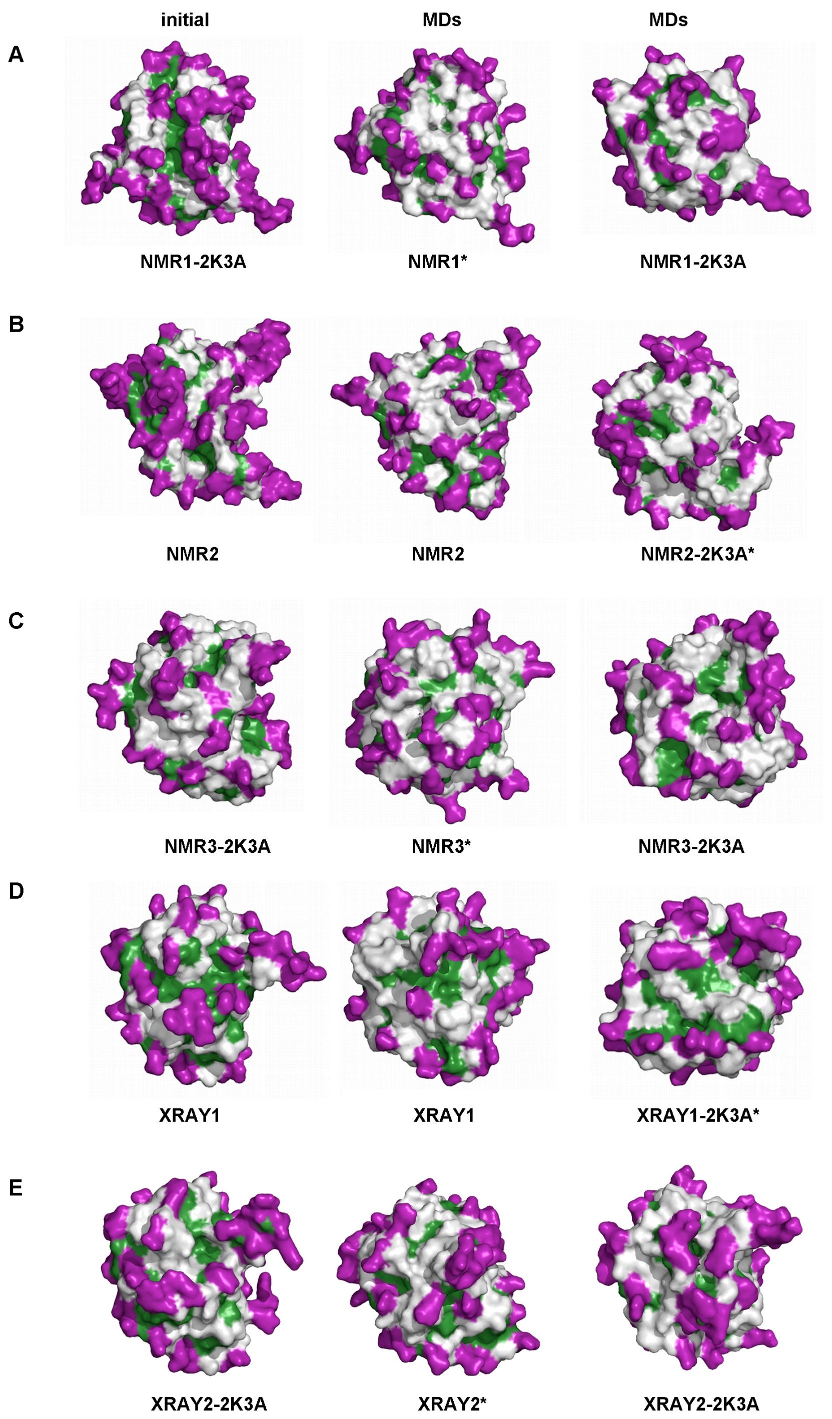

Figure S6. Exposed surface area of the A3G C-CDA. Comparison of the exposed surface area of starting structures with the representative structures from the clustering analysis of MD simulations. Residues indicated in purple have a SASA value greater than 90 Å^2 and those indicated in green have a SASA value lower than 40 Å^2. SASA values were calculated with the POPS program. (A) NMR1-2K3A; (B) NMR2; (C) NMR3-2K3A; (D) XRAY1 and (E) XRAY2-2K3A.
