# Supplementary material for: Rationalisation of the Differences between APOBEC3G Structures from Crystallography and NMR Studies by Molecular Dynamics Simulations
Source: PLoS One. 2010 Jul 12;5(7):e11515. doi: 10.1371/journal.pone.0011515 (PMC2902501; doi:10.1371/journal.pone.0011515)
Supplement: Figure S7 — Positioning of amino acids that mediate interactions of the A3G C-CDA with the DNA substrate. Comparison starting structures with the most representative structure extracted by clustering analysis from the MD simulations as ribbon representations. Amino acid residues R215, E259 and D316 are shown in stick representations and are indicated with the letters R, E and D, respectively. These three amino acids represent the agreement between three independent studies reporting residues within the A3G C-CDA that mediated interactions with the DNA substrate [28]–[30]. The zinc ion at each catalytic core is shown as a grey sphere. (A) NMR1-2K3A; (B) NMR2; (C) NMR3-2K3A; (D) XRAY1 and (E) XRAY2-2K3A. (3.63 MB PDF) [file pone.0011515.s007.pdf]

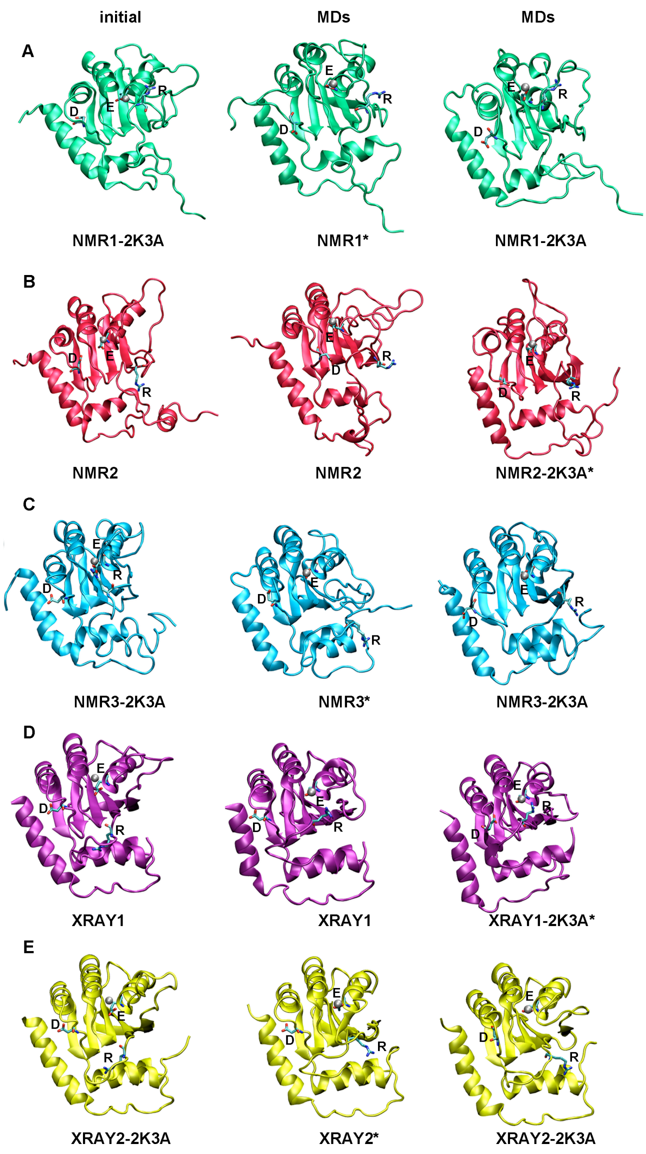

Figure S7. Positioning of amino acids that mediate interactions of the A3G C-CDA with the DNA substrate. Comparison starting structures with the most representative structure extracted by clustering analysis from the MD simulations as ribbon representations. Amino acid residues R215, E259 and D316 are shown in stick representations and are indicated with the letters R, E and D, respectively. These three amino acids represent the agreement between three independent studies reporting residues within the A3G C-CDA that mediated interactions with the DNA substrate [28–30]. The zinc ion at each catalytic core is shown as a grey sphere. (A) NMR1-2K3A; (B) NMR2; (C) NMR3-2K3A; (D) XRAY1 and (E) XRAY2-2K3A.
