# Supplementary material for: Rationalisation of the Differences between APOBEC3G Structures from Crystallography and NMR Studies by Molecular Dynamics Simulations
Source: PLoS One. 2010 Jul 12;5(7):e11515. doi: 10.1371/journal.pone.0011515 (PMC2902501; doi:10.1371/journal.pone.0011515)
Supplement: Table S1 — Percentage of secondary structure in the A3G C-CDA domain before and after simulations. Secondary structure was calculated with the DSSP algorithm for the initial and representative structures from clustering analysis. We performed duplicate simulations with each structure, which are marked as MD1 and MD2 in the table. Simulations described in detail in the text correspond to the data from MD1. (0.15 MB DOC) [file pone.0011515.s008.doc]

| Protein | % α+β  initial | % α+β  MDs | % α  initial | | % α  MDs | % β  initial | % β  MDs |
| --- | --- | --- | --- | --- | --- | --- | --- |
| NMR1*  MD1 | 78 | 77 | 48 | 42 | | 31 | 35 |
| NMR1*  MD2 | 78 | 85 | 48 | 46 | | 31 | 39 |
| NMR1-2K3A  MD1 | 79 | 68 | 48 | 35 | | 31 | 33 |
| NMR1-2K3A  MD2 | 79 | 76 | 48 | 41 | | 31 | 35 |
| NMR2  MD1 | 81 | 73 | 50 | 40 | | 32 | 32 |
| NMR2  MD2 | 81 | 79 | 50 | 42 | | 32 | 37 |
| NMR2-2K3A*  MD1 | 80 | 78 | 48 | 33 | | 32 | 39 |
| NMR2-2K3A*  MD2 | 80 | 73 | 48 | 41 | | 32 | 32 |
| NMR3*  MD1 | 75 | 77 | 43 | 45 | | 32 | 32 |
| NMR3*  MD2 | 75 | 80 | 43 | 45 | | 32 | 35 |
| NMR3-2K3A  MD1 | 79 | 87 | 45 | 46 | | 32 | 41 |
| NMR3-2K3A  MD2 | 79 | 86 | 45 | 46 | | 32 | 40 |
| XRAY1  MD1 | 88 | 87 | 51 | 48 | | 38 | 41 |
| XRAY1  MD2 | 88 | 92 | 51 | 52 | | 38 | 40 |
| XRAY1-2K3A*  MD1 | 88 | 89 | 50 | 47 | | 38 | 41 |
| XRAY1-2K3A*  MD2 | 88 | 89 | 50 | 48 | | 38 | 41 |
| XRAY2*  MD1 | 88 | 87 | 50 | 49 | | 38 | 38 |
| XRAY2*  MD2 | 88 | 87 | 50 | 48 | | 38 | 39 |
| XRAY2-2K3A  MD1 | 88 | 90 | 50 | 50 | | 38 | 41 |
| XRAY2-2K3A  MD2 | 88 | 89 | 50 | 49 | | 38 | 40 |

**Table S1. Percentage of secondary structure in the A3G C-CDA domain before and after simulations.** Secondary structure was calculated with the DSSP algorithm for the initial and representative structures from clustering analysis. We performed duplicate simulations with each structure, which are marked as MD1 and MD2 in the table. Simulations described in detail in the text correspond to the data from MD1.
